# Regulation of Cysteine Homeostasis and Its Effect on *Escherichia coli* Sensitivity to Ciprofloxacin in LB Medium

**DOI:** 10.3390/ijms25084424

**Published:** 2024-04-17

**Authors:** Galina Smirnova, Aleksey Tyulenev, Lyubov Sutormina, Tatyana Kalashnikova, Nadezda Muzyka, Vadim Ushakov, Zoya Samoilova, Oleg Oktyabrsky

**Affiliations:** Institute of Ecology and Genetics of Microorganisms, Perm Federal Research Center, Russian Academy of Sciences, Goleva 13, 614081 Perm, Russia; leksey333@yandex.ru (A.T.); lyubov-sutormina@mail.ru (L.S.); tatyana-kalashnikova22@yandex.ru (T.K.); mu2ykana@mail.ru (N.M.); ushakovvad@yandex.ru (V.U.); samzu@mail.ru (Z.S.); oktyabr@iegm.ru (O.O.)

**Keywords:** cysteine homeostasis, glutathione, H_2_S, Fur and SOS regulons, growth, respiration, antibiotics, bacterial survival

## Abstract

Cysteine and its derivatives, including H_2_S, can influence bacterial virulence and sensitivity to antibiotics. In minimal sulfate media, H_2_S is generated under stress to prevent excess cysteine and, together with incorporation into glutathione and export into the medium, is a mechanism of cysteine homeostasis. Here, we studied the features of cysteine homeostasis in LB medium, where the main source of sulfur is cystine, whose import can create excess cysteine inside cells. We used mutants in the mechanisms of cysteine homeostasis and a set of microbiological and biochemical methods, including the real-time monitoring of sulfide and oxygen, the determination of cysteine and glutathione (GSH), and the expression of the Fur, OxyR, and SOS regulons genes. During normal growth, the parental strain generated H_2_S when switching respiration to another substrate. The mutations affected the onset time, the intensity and duration of H_2_S production, cysteine and glutathione levels, bacterial growth and respiration rates, and the induction of defense systems. Exposure to chloramphenicol and high doses of ciprofloxacin increased cysteine content and GSH synthesis. A high inverse relationship between log CFU/mL and bacterial growth rate before ciprofloxacin addition was revealed. The study points to the important role of maintaining cysteine homeostasis during normal growth and antibiotic exposure in LB medium.

## 1. Introduction

An in-depth study of the adaptive reactions of bacteria to the action of unfavorable factors, including antimicrobial drugs, is necessary when searching for adjuvants that increase the efficiency of existing antibiotics and when creating new drugs. One of the areas that has been actively developing in recent years is the study of the role of cysteine and its derivatives as factors influencing the virulence of bacteria and their sensitivity to oxidative stress and antibiotics [1]. Cysteine is a component of proteins and glutathione and is a source of reduced sulfur for many other organic molecules. Due to a high redox activity of sulfhydryl groups, it plays an important role in the regulation of enzyme activity, cellular signaling, antioxidant defense, and other metabolic processes. However, high concentrations of cysteine in the cytoplasm are dangerous for cells, since, by reducing iron, it increases the pool of Fe^2+^, which, interacting with H_2_O_2_, produces highly toxic hydroxyl radicals during the Fenton reaction [2,3]. In addition, even subtoxic concentrations of cysteine in the cell can inhibit the activity of some enzymes involved in amino acid synthesis, impairing bacterial growth [4]. To prevent these negative consequences, the concentration of free cysteine in the cytoplasm is maintained at a low level (100–200 µM) [2]. Excess cysteine down-regulates its own synthesis by inhibiting the activity of serine transacetylase. Moreover, the product of cysteine degradation H_2_S is an anti-inducer of transcriptional activator CysB, which regulates the synthesis of cysteine from L-serine and the transport of oxidized sulfur sources [5,6]. Other mechanisms of cysteine homeostasis include the export of its excess amounts into the medium, incorporation into glutathione, and degradation with the formation of H_2_S [3,7,8]. *E. coli* cells contain cysteine export systems (EamA, EamB, Bcr, CydDC) [9,10,11] and cystine importers (TcyP and TcyJLN) [3]. Moreover, excess cysteine has been shown to be exported via a primary exporter of the linear neutral class of amino acids AlaE [8]. Several *E. coli* enzymes (CysK, CysM, MetC, TnaA, MalY, and CyuA) exhibit L-cysteine desulfhydrase activity and can degrade cysteine to sulfide and 2-aminoacrilate, which further decomposes to pyruvate and ammonia [12,13,14,15]. L-cysteine aminotransferase (CAT) and 3-mercaptopyruvate sulfurtransferase (3MST or SseA) sequentially metabolize L-cysteine to sulfide and pyruvate [16]. The degradation of cysteine to form H_2_S can also be carried out by cysteine desulfurase (IscS), which catalyzes the conversion of cysteine to alanine and sulfane sulfur [17]. Most of these enzymes are involved in specific metabolic reactions and their role in cysteine degradation remains controversial and manifests itself mainly under conditions of its intracellular excess.

The appearance of excess cysteine in the cytoplasm can occur when cystine or cysteine is added to cells growing in minimal sulfate medium [3,8], or under various starvation stresses and the treatment of *E. coli* with antibiotics (chloramphenicol, tetracycline, ciprofloxacin), causing a sharp inhibition of growth and protein synthesis [7,18,19,20]. Under stress conditions, the disruption of cysteine homeostasis is accompanied by a rapid increase in intracellular reduced glutathione, the release of cysteine into the medium, and transient H_2_S production. The absence of one of these mechanisms of cysteine homeostasis in mutants can be compensated to some extent by other mechanisms when *E. coli* grows in minimal sulfate medium. However, glutathione deficiency in the *gshA* mutant led to an increase in the level of intracellular cysteine under stress, stimulated longer-term sulfide production and significantly increased the sensitivity of this strain to H_2_O_2_ [7,18,19,20]. The protective role of the cysteine–cystine shuttle system, induced in *E. coli* under peroxide stress, was also previously shown [21]. Endogenous and exogenous H_2_S have been found to protect many bacterial species, including pathogens, from oxidative stress and antibiotics with different cellular targets [16,22,23,24,25,26,27,28]. Various mechanisms have been proposed to explain this effect, but further research is needed to more fully understand the processes occurring in cells.

In contrast to minimal sulfate media, where the normal growth of *E. coli* is not accompanied by H_2_S production, in cystine-containing media, H_2_S can be generated by cells regardless of any treatments [29]. This may be a consequence of differences in the maintenance of cysteine homeostasis under conditions where the main source of cysteine is either its synthesis using sulfate or the import of cystine from the environment. Previously, we studied in detail changes in the levels of cysteine, glutathione, and H_2_S under stresses, including the action of antibiotics, in *E. coli* growing in minimal M9 medium [7,9,20]. The purpose of this work was to study the features of cysteine homeostasis in *E. coli* growing in the widely used rich LB medium under normal conditions and under antibiotic-induced stress. We have shown that the disruption of the mechanisms of cysteine homeostasis in mutants causes complex changes in the rate of growth and respiration, the content of low molecular weight thiols and iron, and the activity of defense systems, which ultimately affects lethal activity of ciprofloxacin.

## 2. Results

### 2.1. Effect of Mutations in Cysteine Homeostasis on H_2_S Production in the Absence and Presence of Ciprofloxacin

Previously, we showed that the appearance of H_2_S in liquid LB medium and in gas phase in wild-type *E. coli* was recorded at OD_600_ of about 0.35 and, apparently, was associated with metabolic rearrangement when switching to another substrate in a multicomponent LB medium [29]. We hypothesized that under these conditions, H_2_S production may be a consequence of an excess of intracellular cysteine and triggering of its homeostasis mechanisms.

**Figure 1:**
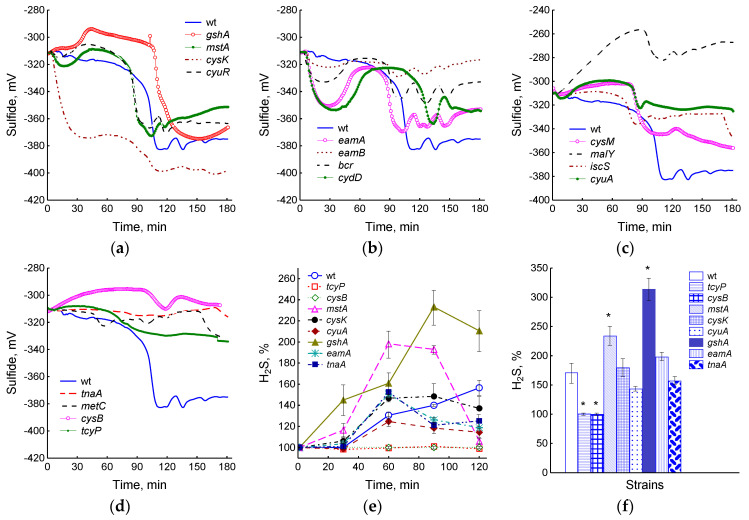
Mutations in cysteine synthesis and degradation *(cysK*, *cysM*, *cyuA*, *iscS*, *malY*, *metC*, *mstA*, *tnaA*), cystine import and cysteine export (*tcyP*, *eamA*, *eamB*, *bcr*, *cydD*), glutathione synthesis (*gshA*), and regulatory proteins (*cyuR* and *cysB*) influenced the intensity and kinetics of sulfide generation in the medium (**a**–**d**) and the accumulation of H_2_S in the gas phase (**e**,**f**). (**a**–**d**) Readings of the sulfide electrode from the moment of inoculation of bacteria into a fresh LB medium. Representative results from one of at least three independent experiments are presented. (**e**,**f**) Data were obtained using lead acetate-soaked paper strips in the OD_600_ range from 0.3 to 1.2 and are shown as the means and standard error (vertical bars) for every 30 min (**e**) or for a total of 2 h (**f**) of incubation. Statistical differences compared to the wild-type strain (*p* < 0.05) are noted with asterisk.

In this work, we determined the production of H_2_S in mutants with disturbances in cysteine homeostasis, including those defective in cysteine synthesis and degradation (*cysK*, *cysM*, *cyuA*, *iscS*, *malY*, *metC*, *mstA*, *tnaA*), in cystine import and cysteine export (*tcyP*, *eamA*, *eamB*, *bcr*, *cydD*), in glutathione synthesis (*gshA*) and in regulatory proteins (*cyuR* and *cysB*). The monitoring of sulfide concentrations in a liquid medium was carried out using a sulfide electrode with a sensitivity threshold of 10 nM. To monitor H_2_S in the gas phase, paper strips soaked with lead acetate were used (sensitivity from 0.1 μM). All the mutations studied affected the intensity and kinetics of H_2_S generation to one degree or another (Figure 1a–d). The *cysK* mutant intensively produced sulfide within 40 min after the bacteria were transferred to fresh medium, then production slowed down and resumed again at the 80th minute, which coincided with the onset of H_2_S production in the wild-type strain (Figure 1a). The total value of the drop in the potential of the sulfide sensor in this mutant was 73 ± 13 mV, which corresponded to the 400–950 nM sulfide. The *cyuR* and *mstA* strains began to form sulfide 20 min earlier and the *gshA* 20 min later than the parent. The amount of H_2_S produced was 250–350 nM (54 ± 4 mV) in the parent and *cyuR* and 450–650 nM in *mstA* (68 ± 6 mV) and *gshA* (67 ± 3 mV) mutants. Mutants deficient in the cysteine export (*eamA*, *eamB*, *bcr*, and *cydD*) represented a special group (Figure 1b). These mutants exhibited multiple cycles of sulfide production, which were recorded as reversible drops in electrode potential. The first cycle began immediately after the bacteria were transferred to fresh medium and was most pronounced in the *eamA* and *cydD* mutants, which released 150 and 180 nM sulfide, respectively. The beginning of the second cycle of H_2_S production in all mutants, with the exception of *cydD*, approximately coincided with the parental strain. The amount of sulfide released was about 250, 90, and 20 nM for *eamA*, *bcr*, and *eamB*, respectively. The *cydD* mutant began to release sulfide 30 min later and produced 180 nM H_2_S. Subsequent cycles were repeated at 20–30 min intervals and were less pronounced. The *cysM*, *malY*, *iscS*, and *cyuA* mutants showed reduced sulfide production: 160, 80, 80, and 60 nM, respectively (Figure 1c). Strains *tnaA*, *metC*, *cysB*, and *tcyP* produced very little or no sulfide (Figure 1d).

Nine strains representing different groups were selected for more detailed studies: *wt*, *gshA*, *tcyP*, *cysB*, *mstA*, *cysK*, *cyuA*, *eamA*, and *tnaA*. When these cultures grew in the OD_600_ range from 0.3 to 1.2, the greatest amount of H_2_S in the gas phase was accumulated by the *gshA* and *mstA* mutants, and the least by *cysB* and *tcyP* (Figure 1e,f and Figure S1). With increasing culture density, the level of H_2_S in the *eamA*, *cyuA*, and *tnaA* mutants decreased relative to the value in the parent (Figure 1e), but the total accumulation of H_2_S over 2 h did not differ significantly (Figure 1f). In general, although the information obtained using the sulfide electrode is more complete, it agrees well with data on the accumulation of H_2_S in the gas phase (r = 0.81, *p* < 0.05).

**Figure 2:**
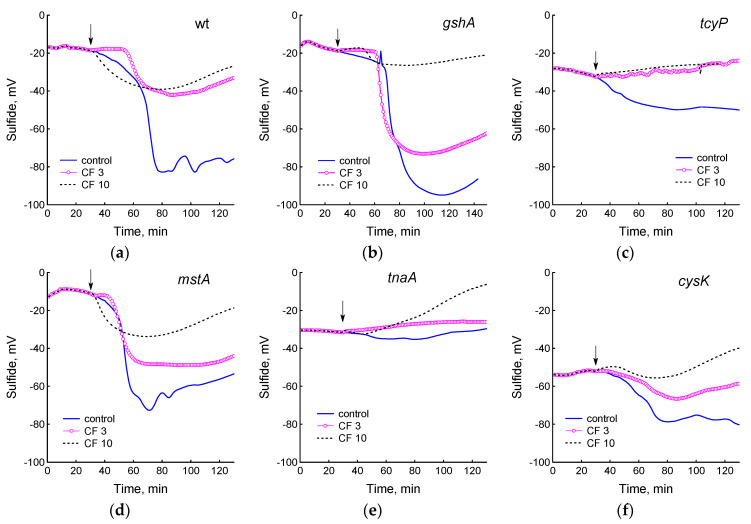

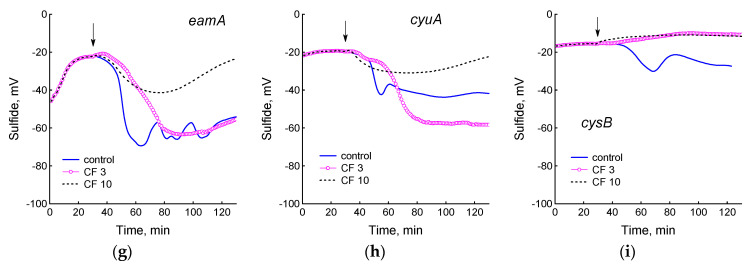
Effect of 3 and 10 μg/mL ciprofloxacin on sulfide production in cultures of the studied *E. coli* mutants. Sulfide electrode readings for wt (**a**), *gshA* (**b**), *tcyP* (**c**), *mstA* (**d**), *tnaA* (**e**), *cysK* (**f**), *eamA* (**g**), *cyuA* (**h**), and *cysB* (**i**) strains are shown. The arrows indicate the moment of adding the antibiotic. Representative results from one of at least three independent experiments are presented.

We have previously shown that the treatment of *E. coli* growing in minimal M9 medium with chloramphenicol or doses of ciprofloxacin exceeding the optimal bactericidal concentration (OBC) causes the generation of H_2_S [7,18]. The addition of these antibiotics to wild-type cells in LB medium in the phase preceding the onset of H_2_S production modified changes in the sulfide sensor potential, affecting its amplitude and duration depending on the concentration of the antibiotic [29]. In this work, we studied how ciprofloxacin (CF) at concentrations corresponding to OBC (3 μg/mL) and exceeding it (10 μg/mL) affects H_2_S production in cysteine homeostasis mutants growing in LB. When cells were treated with 3 and 10 μg/mL CF, sulfide production decreased in most strains, including wt, indicating a slowdown in the processes generating H_2_S (Figure 2). The sulfide electrode potential was reduced two-fold in the wild-type strain at both CF concentrations. In the *cysB*, *tcyP*, and *tnaA* strains, sulfide formation ceased completely. The *gshA*, *mstA*, *eamA*, and *cyuA* mutants continued to produce sulfide at levels close to the control at 3 μg/mL CF, but reduced its production by two to four times at 10 μg/mL CF.

### 2.2. Effect of Mutations in Cysteine Homeostasis on Changes in Intra- and Extracellular Glutathione

Incorporation into glutathione is one of the main mechanisms for the removal of excess cysteine during the stress-induced disturbances of its homeostasis in *E. coli* growing in minimal media [7,20]. In this work, we studied the role of glutathione in maintaining cysteine homeostasis during normal growth and under the influence of antibiotics in LB medium.

**Figure 3:**
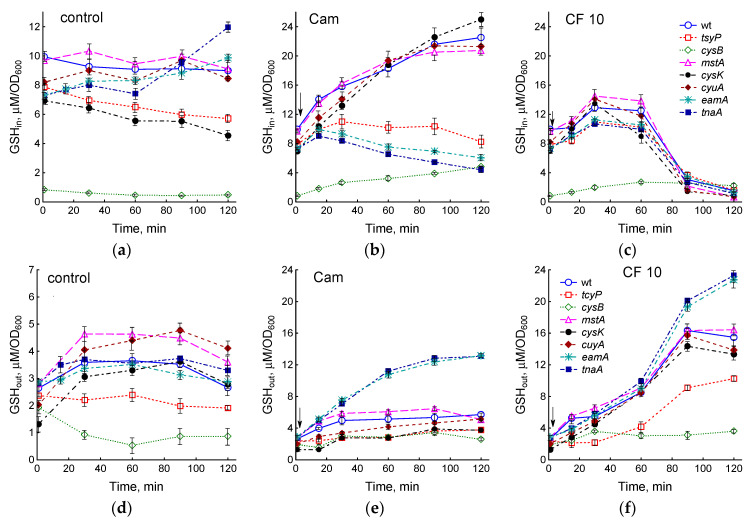
Effect of mutations in cysteine homeostasis on changes in intracellular (**a**–**c**) and extracellular (**d**–**f**) glutathione during normal growth (**a**,**d**) and when *E. coli* is treated with 25 μg/mL chloramphenicol (**b**,**e**) or 10 μg/mL ciprofloxacin (**c**,**f**). The arrows indicate the moment of adding antibiotics (at OD_600_ 0.4). Values are the means and standard error (vertical bars) from at least three independent experiments.

Intracellular GSH (GSH_in_) in the wt strain, amounting to 9.9 ± 0.3 μM/OD_600_, remained at an approximately constant level during cultivation (Figure 3a). The GSH_in_ concentration in *mstA* and *cyuA* mutants was close to that in the wt strain. The *cysB* mutant had the minimum level of glutathione (0.84 ± 0.03 μM/OD_600_); in the *tcyP*, *cysK*, and *tnaA* strains, the GSH_in_ content was 1.3–1.4 times lower than in the parent. Unlike other strains, the GSH_in_ level in the *tnaA* mutant elevated during cultivation.

Treatment with chloramphenicol (Cam) caused an increase in GSH_in_ in the wt strain to 22.5 ± 1.2 μM/OD_600_ (2.3 times the initial value) (Figure 3b and Figure S2a). The *mstA*, *cyuA*, and *cysK* mutants showed changes in GSH_in_ which were close to those in the parent (Figure 3b). At a low initial value in the *cysB* mutant, Cam caused a 5.7-fold increase in GSH_in_. A minimal increase in the concentration of intracellular glutathione was observed in the *tcyP*, *eamA*, and *tnaA* mutants (1.4, 1.35, and 1.2 times, respectively). In contrast to the other strains, the level of GSH_in_ in *tnaA* and *eamA* mutants decreased after a slight increase (Figure 3b).

A dose of 0.3 μg/mL ciprofloxacin had no effect on GSH_in_ levels, but doses of 3 and 10 μg/mL CF increased GSH_in_ 1.4-fold (Figure S2a). After 90 min at 3 μg/mL CF and after 60 min at 10 μg/mL CF, a rapid decrease in GSH_in_ was observed. All studied mutants showed similar kinetics of changes in GSH_in_ under the action of ciprofloxacin (Figure 3c). When treated with 10 μg/mL CF, the maximum concentration of GSH_in_ (1.3–1.9 times higher than the initial one) was reached after 30 min; after 60 min of exposure, the glutathione level began to decrease, approaching zero after 2 h. The exception was the *cysB* mutant, which did not exhibit a significant drop in GSH_in_.

Part of the glutathione synthesized by cells is exported to the medium during aerobic growth of *E. coli*. Because LB medium itself contains glutathione (11.2 ± 0.6 μM in our assays), the fraction of GSH released by bacteria (GSH_out_) was determined by subtracting the glutathione contained in LB from its total concentration in the medium. At OD_600_ 0.4, the GSH_out_ level in different strains ranged from 1.3 to 2.8 μM/OD_600_ and in most cases increased by 1.3–2.8 times with increasing biomass (Figure 3d). In the *tcyP* mutant, GSH_out_ did not change, while in *cysB* it decreased during bacterial cultivation.

When treated with chloramphenicol, simultaneously with the accumulation of GSH inside the cells, part of glutathione was released into the medium (Figure 3e). In most strains, including wt, the GSH_out_ level increased two to three times relative to the initial values. The minimum increase in GSH_out_ (1.6 times) was shown by the *tcyP* mutant, the maximum (4.6 times) by the *tnaA* and *eamA* mutants, which intensively exported GSH into the medium, preventing its accumulation in the cytoplasm (Figure 3b,e).

Ciprofloxacin stimulated GSH accumulation in the medium in a dose-dependent manner in the wild-type strain (Figure S2b). At 10 µg/mL CF, the increase in GSH_out_ began earlier and was more intense than at 3 µg/mL; at 0.3 μg/mL CF, the GSH concentration in the medium did not change. The increase in GSH_out_ occurred synchronously with the decrease in GSH_in_, indicating the release of glutathione from the cells. In all the mutants studied, with the exception of *cysB*, a sharp acceleration in the release of GSH from cells and its accumulation in the medium were observed 60 min after the addition of 10 μg/mL CF (Figure 3f). As with Cam treatment, the *tcyP* mutant accumulated less and *tnaA* and *eamA* more extracellular GSH compared to the other strains. GSH_out_ in the *cysB* mutant remained at a low level despite CF addition. Quantitatively, the decrease in GSH_in_ approximately corresponded to the increase in GSH_out_ in all strains except *tnaA* and *eamA*, which was released into the medium two times more GSH. The total amount of glutathione (GSH_in_ + GSH_out_) synthesized by cells upon exposure to 10 μg/mL CF increased by 1.5 times in strains wt, *mstA*, *cysK*, and *cyuA*; 2 times in *cysB*, *tnaA*, and *eamA*; and 1.25 times in *tcyP* compared to control. As a result of treatment with chloramphenicol, total glutathione increased by 1.4 times in *tcyP*, 2.6 times in *cysB*, and 2.9 times in *cysK* mutants. In other strains, including wt, the total amount of GSH increased by approximately two times. Thus, chloramphenicol and ciprofloxacin stimulated GSH synthesis, the most likely reason for which is the occurrence of excess intracellular cysteine due to the inhibition of protein synthesis and other processes that consume cysteine.

### 2.3. Effect of the Studied Mutations on the Level of Intracellular Cysteine during Normal Growth and Exposure to Antibiotics

**Figure 4:**
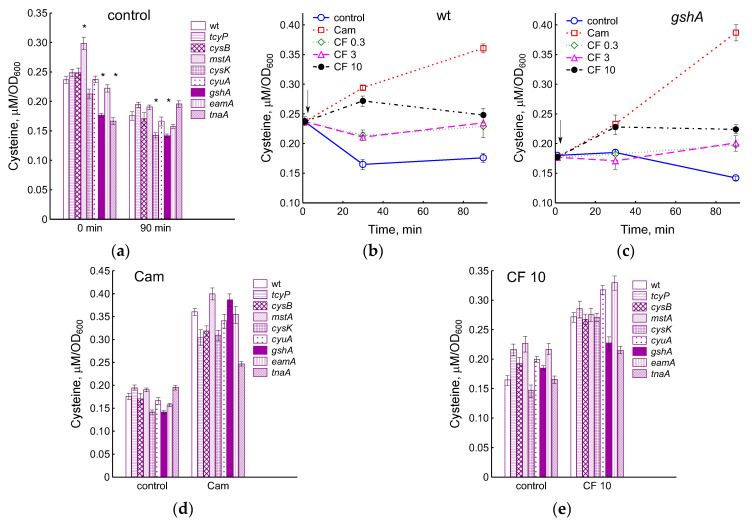
Changes in the concentration of cysteine in the cells of the studied mutants during normal growth in LB medium (**a**) and under the influence of ciprofloxacin (**b**–**e**) and chloramphenicol (**d**). (**b**,**c**) Exposure of wt and *gshA* strains to CF (0.3–10 μg/mL) and Cam (25 μg/mL). (**d**) Cysteine level in cells 90 min after Cam treatment. (**e**) Cysteine level in cells 30 min after treatment with 10 μg/mL CF. The arrows indicate the moment of adding antibiotics (at OD_600_ 0.4). Means and standard errors (vertical bars) from at least three independent experiments are presented. Statistical differences compared to the wild-type strain (*p* < 0.05) are noted with asterisk.

The inability to incorporate excess cysteine into glutathione (*gshA*), reduced import of cystine into the cytoplasm and export of cysteine into the environment (*cysB*, *tcyP*, *eamA*), as well as the absence of enzymes for the degradation and synthesis of cysteine (*mstA*, *cyuA*, *tnaA*, *cysK*) may be the cause of disturbances intracellular cysteine homeostasis. We measured intracellular cysteine concentrations in these mutants during normal growth and under antibiotic treatment. In the exponential phase, intracellular concentration of cysteine in the *mstA* mutant was 1.26 times higher, and in the *gshA* and *tnaA* mutants it was 1.34 and 1.42 times lower than that in the parent (Figure 4a). In all strains, with the exception of *tnaA*, the cysteine content decreased during cultivation from 1.25 to 1.5 times, while after 90 min the *gshA* and *cysK* mutants had a lower cysteine level than the parent.

We have previously shown that triggering the mechanisms of intracellular cysteine homeostasis when treating *E. coli* with chloramphenicol or ciprofloxacin in M9 medium prevent an increase in the concentration of cysteine in wild-type cells but not in the *gshA* mutant [7]. Unlike M9, where sulfate is used for cysteine synthesis, in LB medium, the supply of cysteine to *E. coli* cells is carried out by the transport of cystine with two CysB-controlled importers, the main of which is GSH-dependent TcyP (YdjN) [3]. The exposure of *E. coli* to chloramphenicol in LB medium led to an increase in the level of intracellular cysteine by 2 times relative to the control in the parental strain and by 2.7 times in the *gshA* mutant (Figure 4b,c). A statistically significant increase (*p* < 0.05) in intracellular cysteine in response to chloramphenicol was also observed in all mutants studied (Figure 4d). Under these conditions, cysteine level increased by 1.55-fold in the mutant lacking cystine transporter TcyP and by 2.25-fold in the mutant deficient in cysteine exporter EamA (Figure 4d). The smallest increase in cysteine content (1.26 times) was observed in the *tnaA* mutant with an impaired ability to produce H_2_S in LB medium. Changes in intracellular cysteine under the influence of chloramphenicol in other strains were close to the parent.

During exposition to 0.3 and 3 μg/mL CF, the concentration of intracellular cysteine in the wt and *gshA* strains did not change, but due to a decrease in the control level after 90 min of exposure, it was 1.3 and 1.4 times higher than that in the control (Figure 4b,c). A statistically significant increase in cysteine concentration by 15 and 30% in wt and *gshA* cells, respectively, was observed 30 min after treatment with 10 μg/mL CF. Cysteine levels in the cells of all studied strains under these conditions exceeded the corresponding control by 1.65 (wt), 1.23 (*gshA*), 1.32 (*tcyP*), 1.39 (*cysB*), 1.22 (*mstA*), 1.84 (*cysK*), 1.59 (*cyuA*), 1.52 (*eamA*), and 1.3 (*tnaA*) times (Figure 4e). Thus, the treatment of cells with a high dose of CF affected cysteine homeostasis in all strains, although to varying degrees.

### 2.4. Changes in the Expression of Antioxidant Genes in the Studied Mutants

**Figure 5:**
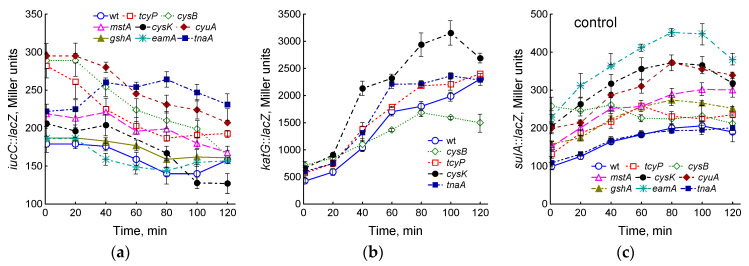

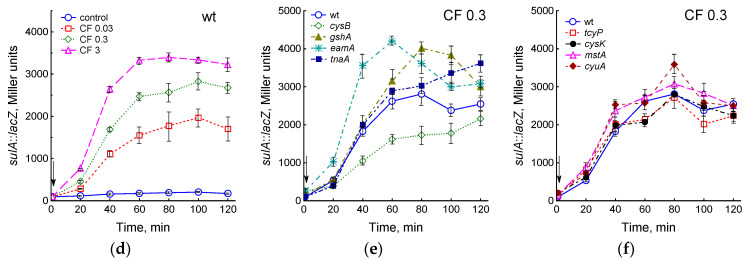
Effect of the studied mutations on the expression of *iucC*::*lacZ* (**a**), *katG*::*lacZ* (**b**) and *sulA*::*lacZ* (**c**–**f**) during the normal growth of *E. coli* in LB medium (**a**–**c**) and when treating with CF (**d**–**f**). (**d**) Expression of *sulA*::*lacZ* upon exposure of the parental strain to ciprofloxacin (0.03–3 μg/mL). (**e**,**f**) Expression of *sulA*::*lacZ* when the studied mutants were treated with 0.3 μg/mL CF. The arrows indicate the moment of adding CF (at OD_600_ 0.4). Means and standard errors (vertical bars) from at least three independent experiments are presented.

Increasing cysteine concentration poses a risk of oxidative stress as it may be a source of ROS and serve as a reducer of Fe^3+^ to Fe^2+^ in the cytoplasm, promoting the Fenton reaction between H_2_O_2_ and Fe^2+^ [2,3,8]. To assess changes in free iron and H_2_O_2_ levels in mutant cells with impaired cysteine homeostasis mechanisms, we measured the expression of *iucC*::*lacZ* and *katG*::*lacZ* fusions in the studied strains. The *iucC* gene encodes aerobactin and is under the control of the transcriptional regulator Fur, which represses its regulon after binding with [2Fe–2S] cluster [30]. The degree of *iucC*::*lacZ* expression may indirectly indicate the level of free iron in the cytoplasm [31]. A decrease in intracellular free iron stimulates *iucC* expression. The *cysB*, *tcyP*, and *cyuA* mutants showed 1.6-fold and the *mstA* and *tnaA* mutants showed 1.2-fold higher levels of *iucC*::*lacZ* expression than the wt strain (Figure 5a), which may indicate lower free iron content compared to the parent. Other strains studied had *iucC*::*lacZ* expression close to the wt strain. The expression of *iucC*::*lacZ* in the *tnaA* mutant increased during cultivation and after 80 min was 1.9 times higher than that in the parent. In all other mutants, *iucC*::*lacZ* expression gradually decreased.

The expression of the *katG* gene, encoding catalase HPI, can be regulated by the transcription regulator OxyR, which is activated by an increase in H_2_O_2_, and by the regulator of the general stress response RpoS during the transition to the stationary phase [32]. The expression of *katG*::*lacZ* in exponentially growing (OD_600_ 0.4) mutants *tcyP*, *cysB*, *cysK*, and *tnaA* was 1.35, 1.7, 1.55, and 1.4 times higher than that in the parent, respectively (Figure 5b). An increase in culture density was accompanied by the induction of the *katG* gene in all studied strains. The *cysK* and *tnaA* mutants maintained a higher *katG*::*lacZ* expression than wt throughout the experiment. Other mutants did not differ significantly from the parental strain at all growth phases (Figure S3).

Hydroxyl radicals produced in the Fenton reaction can damage all biological molecules, including DNA. In response to DNA damage, *E. coli* induces the SOS regulon, which controls genes involved in DNA repair [33]. One of the SOS-controlled genes is the filamentation mediator *sulA*. We used the *sulA*::*lacZ* transcriptional fusion to assess the degree of the induction of the SOS response in the mutants studied. All mutants except *tnaA* showed 1.3- to 2.3-fold higher levels of *sulA*::*lacZ* induction, indicating an increased potential for DNA damage in these strains (Figure 5c). The expression of *sulA* increased during cultivation in all strains, with the exception of *cysB*.

Fluoroquinolone ciprofloxacin kills bacteria by damaging their DNA through direct binding with DNA gyrase and/or topoisomerase IV, which results in the formation of double-strand DNA breaks (DSBs), replication arrest, SOS response induction, chromosome fragmentation, and cell death [34]. Consistent with this, ciprofloxacin induced *sulA*::*lacZ* expression in the cells of the wt strain in a dose-dependent manner (Figure 5d). Treatment with 0.3 μg/mL CF caused an increase in *sulA* expression in all mutants studied (Figure 5e,f). The greatest differences from the parent were observed in the *eamA*, *gshA*, and *tnaA* mutants, the expression of *sulA*::*lacZ* in which was 1.6, 1.4 and 1.4 times higher than in wt, respectively. Interestingly, the maximum expression of *sulA* in these strains was reached at different times (60, 80, and 120 min, respectively). In contrast, in the *cysB* mutant, *sulA*::*lacZ* expression was 1.6-fold lower than in the parent, which may indicate a lower degree of DNA damage in this strain.

### 2.5. Consequence of the Studied Mutations on the Physiological Parameters of E. coli and Ciprofloxacin Sensitivity

**Figure 6:**
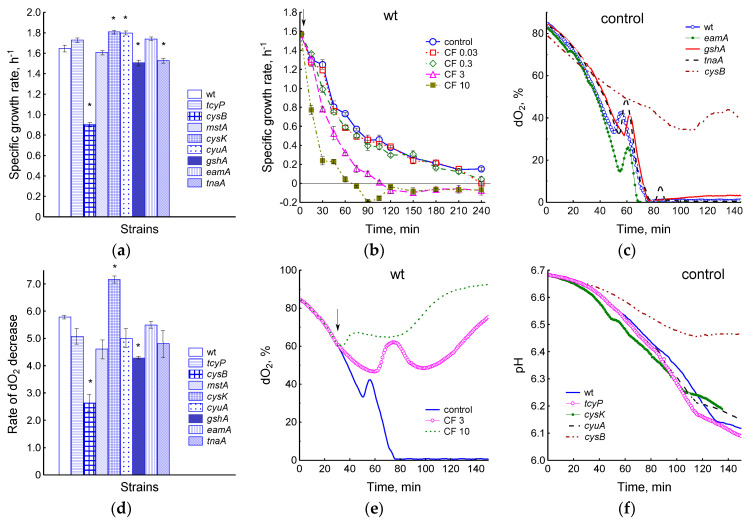
Effect of the studied mutations on the specific growth rate (**a**,**b**), oxygen consumption (**c**–**e**), and pH (**f**) during normal growth (**a**,**c**,**d**,**f**) and exposure to CF (**b**,**e**). (**a**) Value of µ at OD_600_ 0.4. (**b**) Changes in specific growth rate upon exposure of the parental strain to ciprofloxacin (0.03–3 μg/mL). (**c**) Changes in dO_2_ indicates the presence of a transient process. (**d**) Rate of dO_2_ decrease in cultures of the studied mutants. (**e**) Changes in dO_2_ upon exposure of the parental strain to 3 and 10 μg/mL CF. (**f**) pH changes during growth of the studied mutants in LB medium. (**a**,**b**,**d**) Means and standard errors (vertical bars) from at least three independent experiments are presented. Statistical differences compared to the wild-type strain (*p* < 0.05) are noted with asterisk. (**c**,**e**,**f**) Representative results of one of at least three independent experiments are presented. The arrows indicate the moment of adding CF.

Differences in the levels of low molecular weight thiols and in the degree of induction of cellular antioxidant systems may influence physiological parameters such as growth and respiration, as well as the sensitivity of bacteria to antibiotics. The minimum inhibitory concentrations of ciprofloxacin for the *gshA* and *cysB* mutants were 0.064 μg/mL; for the remaining strains, including the parent, it was 0.032 μg/mL. The maximum specific growth rate of the parental strain was 1.65 ± 0.03 h^−1^. The *cysB*, *gshA*, and *tnaA* mutants grew more slowly, while the *cysK* and *cyuA* mutants grew faster than the parent (Figure 6a). In antibiotic-free medium, μ in all strains gradually decreased to 0.1–0.2 h^−1^ within 4 h of incubation (Figure 6b and Figure S4a). Treatment with ciprofloxacin caused growth inhibition proportional to the antibiotic dose used in all *E. coli* strains studied. The addition of 0.03 and 0.3 μg/mL CF in the wt strain had little effect on the change in specific growth rate compared to the control (Figure 6b). CF doses of 3 and 10 μg/mL led to a sharper drop in μ, which took negative values 105 and 70 min after the start of antibiotic exposure, respectively, indicating partial cell lysis. Although the kinetics of changes in μ at different CF concentrations were generally similar for all strains, some quantitative differences were observed (Figure S4). Thus, at a lower initial growth rate, the *gshA* mutant maintained higher μ values, and the *tnaA* mutant maintained lower μ values compared to other strains when incubated with CF. Accordingly, zero μ level upon treatment with 3 and 10 μg/mL CF was achieved after 120 and 75 min in *gshA* and after 85 and 60 min in the *tnaA* mutant (Figure S4d,e). In the *cysB* mutant, the transition of μ through 0 occurred after 90 min for both CF concentrations.

The decrease in µ occurred in parallel with the decrease in oxygen concentration. A feature of the LB medium, which was observed in all studied strains, was a sharp reversible inhibition of respiration and a corresponding surge in oxygen content when dO_2_ reached 30–40% (Figure 6c). The observed phenomenon may be a reflection of the transition of respiration to another energy substrate in the multicomponent LB medium. The timing of the transition process in different strains seemed to depend on the rate of depletion of the primary substrate and was either ahead of the wt strain in the *cysK* and *cyuA* mutants, or slightly behind, as in *gshA*, *tnaA*, *eamA*, and *cysB* (Figure 6c). The discrepancy with the parent usually did not exceed 10 min; in the slowest growing *cysB* strain it was about 1 h. The rate of decrease in dO_2_, expressed as dO_2_/OD_600_∙min, reflects the rate of cell respiration (Figure 6d). Measured over the time before the onset of the transition process, it was 5.79 ± 0.07 (wt), 2.64 ± 0.3 (*cysB*), 4.29 ± 0.05 (*gshA*), 4.62 ± 0.35 (*mstA*), 4.82 ± 0.61 (*tnaA*), 5.0 ± 0.35 (*cyuA*), 5.08 ± 0.29 (*tcyP*), 5.5 ± 0.1 (*eamA*), and 7.17 ± 0.11 (*cysK*). The respiration rate of the studied mutants was closely related to their growth rate (r = 0.85, *p* < 0.05).

A dose of 0.3 μg/mL CF had little effect on the change in dO_2_ in the parental strain. The addition of 3 μg/mL CF inhibited cellular oxygen consumption and delayed the reversible increase in dO_2_ observed in control cultures and associated with the transition to another substrate by an average of 15 min (Figure 6e and Figure S5). After approximately 60 min, a gradual increase in dO_2_ began, indicating the inhibition of cell respiration. The transient process was absent in the *cysB* mutant. The addition of 10 μg/mL CF in all strains caused a sharp inhibition of respiration, which was biphasic. The beginning of the second phase coincided with the transition of μ to values below 0 and, as we showed earlier, depended on the induction of the SOS response [35].

Within 3 h after bacterial inoculation, there was a gradual decrease in pH due to accumulation of acidic byproducts, followed by an increase in pH, which could be associated with a shift to using amino acids instead of sugars as an energy source. The *tcyP*, *cysK*, and *cyuA* mutants decreased pH slightly faster, and the *cysB* mutants decreased pH much more slowly than the parental strain, which is in good agreement with the growth rate of these bacteria (Figure 6f). Ciprofloxacin dose-dependently suppressed the decrease in pH, which may serve as a marker of energy substrate uptake (Figure S6). Moreover, the studied strains differed in the time at which the acidification of the medium ceased. The pH value stopped decreasing on average 80 min after adding 3 μg/mL CF. The *tnaA* and *tcyP* mutants showed the shortest (55 and 65 min), and *cysK* the longest (90 min) acidification duration. In general, the presence of the studied mutations influenced the duration of the active phase of metabolism, during which growth, respiration, and energy consumption continued in the presence of the antibiotic.

**Figure 7:**
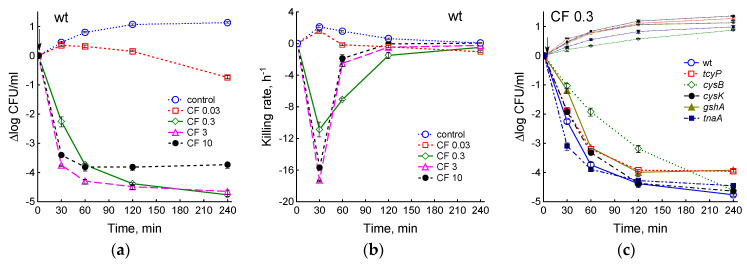

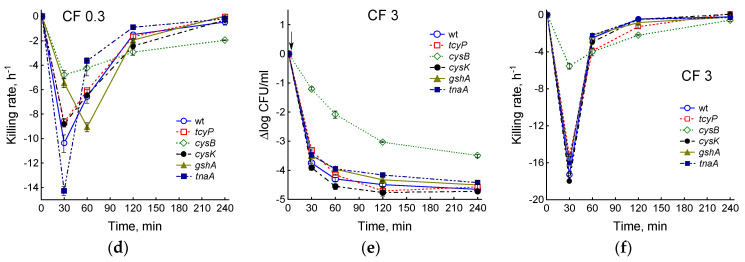
Effect of the studied mutations on the lethal activity of ciprofloxacin. (**a**,**c**,**e**) Killing curves. (**b**,**d**,**f**) Killing rate. (**a**,**b**) Kt strain exposed to 0.03–10 µg/mL CF. (**c**,**d**) Effect of 0.3 μg/mL CF on the studied mutants. (**e**,**f**) Effect of 3 μg/mL CF on the studied mutants. Means and standard errors (vertical bars) from at least three independent experiments are presented. The arrows indicate the moment of adding CF.

We next investigated how mutation-induced changes in physiological parameters and cysteine homeostasis affect the bactericidal activity of ciprofloxacin. By determining CFU, bacterial killing curves were obtained and killing rates were calculated for the ciprofloxacin concentrations of 0.03, 0.3, 3, and 10 μg/mL (Figure 7 and Figure S7). In the parental strain, the maximum bactericidal effect was exerted by a dose of 3 μg/mL CF, which corresponds to the optimal bactericidal concentration in LB medium. Below and above this concentration of the antibiotic, its lethal activity decreased (Figure 7a). The maximum killing rate was observed during the first 30 min after the addition of ciprofloxacin, that is, during the period when the culture maintained a fairly high metabolic activity and growth rate (Figure 7b). In the CF concentration range of 0.3–10 μg/mL, the killing curves reached a plateau and, accordingly, the killing rate decreased to 0, the faster the higher the antibiotic dose. The number of CFU in control cultures of the studied *E. coli* strains was directly proportional to the bacterial growth rate, and after 4 h of cultivation, it was higher in the *cysK* mutant and lower in *cysB* and *tnaA* than in the wt strain (Figure 7c).

A dose of 0.03 μg/mL CF did not cause a decrease in CFU in all strains in the first 60 min of exposure, then slow cell death began (Figure S7a,b). Under these conditions, the number of CFU for each strain, as in the control, was determined mainly by its growth rate. The greatest differences between strains were observed at 0.3 μg/mL CF (Figure 7c,d). At 30 min after adding this dose of CF, the killing rate of the *tnaA* mutant was 1.4 times higher, and that of the *tcyP*, *cysK*, *gshA*, and *cysB* mutants was 1.2, 1.17, 1.9, and 2.2 times lower than at the parent (Figure 7d). However, under conditions of rapid death in the first phase, the *tnaA* mutant quickly reached a plateau corresponding to the number of persisters, and the killing rate decreased. At 1 h after the addition of CF, the death of the *tnaA* mutant occurred 1.8 times slower than the wt strain, while the *gshA* mutant continued to die 1.4 times faster than wt. The killing of the *mstA*, *cyuA*, and *eamA* mutants by 0.3 μg/mL CF was not significantly different from the parental strain (Figure S7c,d). After 4 h of exposure, the highest killing rate was observed in the *cysB* mutant (4 times higher than in the wt strain) (Figure 7d). At 3 and 10 μg/mL CF, differences between strains, with the exception of *cysB*, were less pronounced (Figure 7e,f and Figure S7e–h). The *cysK* mutant, which had the highest growth rate before CF treatment, produced 2 times fewer colonies, and the slower growing *gshA* and *tnaA* formed 2.5 times more colonies than wt when exposed to 3 μg/mL CF (Figure 7e). The killing rate in the *cysB* mutant during the rapid death phase was three and four times lower than in the parent, but after 1 h of exposure to the antibiotic it became three to four times higher due to a sharp inhibition of the death of parental cells (Figure 7f and Figure S7h). Thus, the effect of the studied mutations on lethal activity of ciprofloxacin strongly depended not only on the type of mutation but also on the dose of the antibiotic and exposure time. Strains exhibiting less sensitivity to CF in the first phase maintained a higher killing rate during prolonged exposure to the antibiotic.

## 3. Discussion

*E. coli* produces H_2_S during normal growth in rich LB medium as opposed to minimal medium with sulfate as the sole sulfur source. L-cysteine and L-cystine have been shown to be the most suitable substrates for H_2_S production, while GSH and methionine do not contribute significantly [36]. The monitoring of sulfide using a sulfide electrode with the simultaneous monitoring of dO_2_ made it possible to establish that the onset of H_2_S production in the wild-type strain and most of the mutants studied coincides with a sharp reversible inhibition of respiration. This may indicate a transient process in which an excess of intracellular cysteine occurs, similar to that observed when protein synthesis is inhibited in *E. coli* in minimal medium [7]. The production of H_2_S is the result of the degradation of excess cysteine and one of the mechanisms for maintaining its homeostasis. Studies of sulfide production in mutants with defects in the cystine and cysteine transport systems and glutathione synthesis revealed the complex contribution of various mechanisms to intracellular cysteine homeostasis and their ability to compensate each other. Mutants *eamA*, *eamB*, *bcr*, and *cydD*, which were previously reported to be involved in the export of cysteine from cells to the medium [9,10], cyclically produced H_2_S immediately after transfer to fresh medium and during further cultivation (Figure 1b). The observed behavior of these mutants indicates a requirement for cysteine export to finely regulate cytoplasmic cysteine levels. Increased H_2_S production in the *gshA* mutant confirms the important role of glutathione as a cysteine buffer, as occurs in minimal medium [7].

The generation of H_2_S can be carried out involving enzymes L-cysteine aminotransferase and 3-mercaptopyruvate sulfurtransferase (CAT/MST pathway) [16] or L-cysteine desulfhydrases (CD) (CysK, CysM, MetC, TnaA, MalY, and CuyA) [12,13] and cysteine desulfurase (IscS) [17]. In our experiments, the *mstA* (*sseA*) mutant generated slightly more sulfide than the wt strain. This result contradicts the data of Shatalin et al. [16] but is consistent with the results of other researchers [36,37]. The inconsistency in the data may be due to the appearance in the MST-deficient strain of a compensatory mutation in the transcriptional repressor gene *ycjW*, which has been shown to restore H_2_S formation via the upregulation of the gene encoding rhodanese (thiosulfate sulfurtransferase) PspE [38]. CDs are multifunctional enzymes, and L-cysteine metabolism is usually not their main physiological function. For example, TnaA is better known as tryptophanase that degrades L-tryptophan to indole, whereas the cysteine desulfurase IscS acts as a sulfur donor and is involved in biological sulfur trafficking and the assembly of iron–sulfur clusters, although both can degrade excess cysteine [12,17]. In our studies, deletions of the *malY*, *metC*, *cyuA*, and *iscS* genes significantly reduced the amplitude of changes in the sulfide electrode potential, indicating a decrease in H_2_S generation compared to the wt strain. The *tnaA* mutant showed minimal changes in the electrode potential, but the total accumulation of H_2_S in the gas phase at later stages of cultivation did not reveal a significant difference between the wt, *cyuA*, and *tnaA* strains, which is consistent with earlier data [36]. Although CysK and CysM have CD activity [12], they are better known as H_2_S-consuming cysteine synthases [6]. The sharp and irreversible decrease in sulfide electrode potential upon the inoculation of the *cysK* mutant into fresh medium appears to reflect the inability of this strain to incorporate the resulting H_2_S back into cysteine, which leads to its accumulation in the medium. In contrast to *cysK*, the *cysM* mutant produced significantly less sulfide than the parental strain. We previously showed that the deletion of *cysM* prevents sulfide generation by *E. coli* cells when treated with valine, chloramphenicol, and ciprofloxacin in minimal M9 medium [7,39]. An increase in H_2_S production in the *cysK* mutant and its decrease in the *cysM* during *E. coli* growth in LB medium was noted previously when determining H_2_S with lead acetate and monobromobimane [36]. Slow cystine import in the *tcyP* and *cysB* mutants, which lack, respectively, the main cystine transport system TcyP and the CysB regulator, which controls both cystine importers TcyP and TcyJLN [3], led to an almost complete cessation of sulfide production due to the absence of excess cysteine in the cytoplasm of these strains.

Cysteine limits glutathione synthesis in *E. coli* during growth in minimal medium with sulfate as the only sulfur source [40]. LB medium contains cystine (96.4 ± 1.1 μM cysteine), which is directly imported by cells. However, the limited import of cystine in the *tcyP* and *cysB* mutants and the intense loss of H_2_S in the *cysK* mutant appear to reduce the flux of cysteine to GSH synthesis, resulting in a decrease in its intracellular concentration in these strains. In all strains studied, approximately 20% of the total synthesized GSH (1–3 µM/OD_600_) was released into the medium in addition to the glutathione contained in the LB itself. The concentration of glutathione accumulated by cells in the medium was closely correlated with the level of intracellular GSH (r = 0.89, *p* < 0.05) and with the concentration of intracellular cysteine (r = 0.74, *p* < 0.05).

Despite the lower rate of cystine import, the reduction in GSH synthesis and H_2_S production allowed the *cysB* and *tcyP* mutants to maintain intracellular cysteine at levels close to the parent. The level of cysteine in the *cysK* mutant decreased significantly relative to that in the wt strain only at the late stages of cultivation. Interestingly, the *gshA* mutant maintained a 1.3-fold lower cysteine level compared to the parent throughout the entire cultivation period. This may be due to the intense production of H_2_S in the background of the limited supply of cystine into cells through the TcyP transporter in the absence of GSH [3]. Moreover, in the absence of a major cysteine buffer, maintaining a lower cysteine level in the *gshA* mutant may have adaptive significance.

The danger of high cysteine concentrations is associated with its ability to reduce Fe^3+^ to Fe^2+^, thereby increasing the Fenton-mediated formation of hydroxyl radicals [2]. The highest degree of the induction of the Fur regulon among all the studied mutants was shown by *cysB*, *tcyP*, *cyuA*, and *tnaA*, which may indicate a low free iron pool in their cells. All of these mutants have a reduced ability to produce sulfide. An inverse correlation was found between *iucC*::*lacZ* expression and H_2_S production (r = −0.71 *p* < 0.05). Recently, it was reported that the Fur repressor is activated and therefore inhibits controlled genes, after binding to the [2Fe–2S] cluster [30]. Fur assembles a [2Fe–2S] cluster from intracellular free Fe^2+^ and sulfide that is provided by L-cysteine and cysteine desulfurase IscS when intracellular free iron content is elevated. It can be assumed that the regulation of Fur by [2Fe–2S] clusters allowed the pools of free Fe^2+^ and cysteine in the cytoplasm to be coordinated to prevent formation of hydroxyl radicals generated by Fenton chemistry.

The increased expression of the *katG* gene, encoding HPI catalase, may indirectly indicate an increase in the intracellular level of H_2_O_2_, which reacts with Fe^2+^ in the Fenton reaction. The maximum level of *katG* induction was demonstrated by the *cysK* mutant. This mutant also had an almost two-fold higher level of *sulA*::*lacZ* expression, which may be the result of induction of the SOS regulon in response to increasing oxidative DNA damage. Increased levels of *sulA*::*lacZ* expression in mutants with defects in cysteine export (*eamA*), H_2_S production (*cyuA*, *cysB*, *tcyP*), and GSH synthesis (*gshA*) indicate an important role of all these mechanisms of cysteine homeostasis in protection against DNA damage. The connection between cysteine metabolism and oxidative stress was previously noted in *Salmonella typhimurium*, whose cysteine auxotrophs Δ*cysE* and Δ*cysB* were oxidatively stressed and had increased catalase activity [41].

In general, the presence of cystine in LB medium leads to the fact that *E. coli* cells face the danger of excess intracellular cysteine and are forced to trigger the mechanisms of its homeostasis under normal growth conditions. The absence of any of the mechanisms of cysteine homeostasis can be compensated to a certain extent by the activation of the others, as well as by the induction of other protective systems, including those involved in the regulation of intracellular iron, H_2_O_2_ destruction, and DNA damage repair.

According to the hypothesis proposed by Collins’ group [42,43,44], which has generated considerable debate [45,46,47], the bactericidal activity of antibiotics is associated with the production of hydroxyl radicals due to metabolic changes when antibiotics act on their primary targets. In this case, factors that stimulate the formation of ROS should increase the sensitivity of bacteria to antibiotics. Nudler and co-workers reported that endogenous and exogenous hydrogen sulfide protects bacteria from bactericidal antibiotics and H_2_O_2_, presumably through the H_2_S-mediated sequestration of free iron and the prevention of the Fenton reaction [16,22,27]. The inactivation of enzymes that produce H_2_S makes these bacteria very sensitive to a wide range of antibiotics. The protective role of H_2_S against antibiotics has been confirmed by other researchers, although different mechanisms have been proposed [23,24,25,26,28].

The fluoroquinolone ciprofloxacin, whose effect on *E. coli* was studied in our work, forms a complex with DNA gyrase, which leads to DNA double-strand breaks, chromosome fragmentation, and, ultimately, cell death [34]. It was also noted that the mechanism of lethality of quinolones may be associated with ROS [42,48]. However, in our previous studies, when growing *E. coli* in minimal M9 medium, we did not detect an increase in sensitivity to ciprofloxacin in mutants with defective redox systems of glutathione and thioredoxin. The effect of mutations and various additives that change the redox situation in cells on the survival of bacteria was inversely proportional to their effect on the growth rate [35,39,49,50]. Redox changes that occurred in cells in response to bactericidal ciprofloxacin and bacteriostatic chloramphenicol were of a similar nature. Both antibiotics induced cysteine homeostasis mechanisms, leading to increased glutathione levels inside and outside cells, cysteine export into the medium, and H_2_S release [7,18,19,35]. The accumulation of H_2_S upon exposure to ciprofloxacin was also observed in *Salmonella typhimurium* when the bacteria were grown with sulfate as the sole sulfur source [51].

In this work, we showed that exposure to chloramphenicol and high doses of ciprofloxacin in LB medium also caused disturbances in cysteine homeostasis and triggered the mechanisms that maintain it. The total level of glutathione (GSH_in_ + GSH_out_) after 2 h of exposure of the wt strain to chloramphenicol or 10 μg/mL ciprofloxacin exceeded the value in the control by 2.4 and 1.5 times, respectively. Particularly, the active incorporation of cysteine into GSH occurred in mutants with low initial levels of glutathione *cysB* and *cysK*, where the total glutathione exceeded the control level by 5.5 and 3.9 times when exposed to Cam and by 4.3 and 2 times when treated with CF, respectively. Because LB medium contains significant amounts of cystine, we were unable to determine whether the release of cysteine from cells is stimulated by antibiotics. An important difference from the minimal medium with sulfate was a decrease in H_2_S production, up to its complete inhibition in a number of mutants, upon exposure to antibiotics in the LB medium compared to the level of sulfide in the control culture. Finally, in LB medium, the intracellular cysteine concentration increased in response to Cam and 10 μg/mL CF in all strains studied, including the parent. The maximum increase in cysteine content under the influence of chloramphenicol was demonstrated by the *gshA* mutant. In contrast, the level of cysteine did not change in the wt strain when exposed to CF and Cam but increased in the *gshA* mutant when *E. coli* cells were grown in minimal medium with sulfate [7]. The lack of glutathione in the *gshA* mutant, as well as the reduced ability to export cysteine in *eamA*, stimulated ciprofloxacin-induced *sulA*::*lacZ* expression compared to the wt strain. This may indicate an additional contribution of oxidative DNA damage caused by excess cysteine to the induction of the SOS regulon, as well as the important role of glutathione as a cysteine buffer in the regulation of its intracellular level.

Overall, the mutants differed from each other in a number of ways, including the ability to produce H_2_S, regulate cysteine homeostasis, induce antioxidant defense systems, and maintain metabolic activity in the presence of ciprofloxacin, which could influence their sensitivity to antibiotics. However, only *gshA* and *cysB* mutants had two-fold higher MICs for ciprofloxacin. Sensitivity to antibiotics is largely determined by the physiological state of the bacteria. Among all the mutations studied, the deletion of the *cysB* gene, which reduced µ by 0.75 h^−1^, had the greatest effect on the rate of bacterial growth and respiration. The specific growth rate of other mutants in the exponential phase was approximately 0.15 h^−1^ higher (*cysK*, *cyuA*) or lower (*gshA*, *tnaA*) than that of the parent. Treatment with ciprofloxacin caused the inhibition of growth, respiration, and energy substrate consumption, proportional to the antibiotic dose used in all studied *E. coli* strains. Within 1 h after treatment, there was a high direct correlation between the specific growth rate values before and after exposure to all doses of ciprofloxacin (r = 0.88, 0.91, 0.79, and 0.82, *p* < 0.05, for 0.03, 0.3, 3, and 10 μg/mL, respectively). There was also a direct relationship (r = 0.81 and r = 0.82, *p* < 0.05) between growth rate after antibiotic exposure and *sulA*::*lacZ* expression and an inverse relationship (r = −0.80 and r = −0.71, *p* < 0.05) between the growth rate and *katG*::*lacZ* expression at CF doses of 0.03 and 0.3 μg/mL, respectively. That is, induction of the SOS regulon contributed to the maintenance of a higher growth rate under the influence of low concentrations of the antibiotic, and *katG* expression was induced when growth slowed down. It should be noted that an increase in the expression of the *katG* gene can occur independently of the activation of the H_2_O_2_-sensitive OxyR during the transition of *E. coli* to the stationary phase under the control of RpoS [32]. When incubated with ciprofloxacin, the *gshA* mutant showed higher specific growth rates, and the *tnaA* mutant showed lower μ values compared to other strains. In general, the presence of the studied mutations influenced the duration of the active metabolic phase, during which bacteria are most vulnerable to the action of antibiotics, including ciprofloxacin [52].

Even small differences in growth rates can significantly alter the survival of bacteria when exposed to antibiotics. Thus, we previously showed that, regardless of the reason, an increase in the growth rate (μ) by 0.1 h^−1^ led to an increase in the ciprofloxacin-induced killing rate (ψ) by about 1 h^−1^, and a two-fold decrease in μ was accompanied by an increase in log CFU/mL by two orders of magnitude [50]. Here we also found a strong inverse relationship between log CFU/mL under the influence of 0.3, 3, and 10 μg/mL CF and the specific growth rate of bacteria before exposure to the antibiotic (after 60 min r = −0.80, −0.96, and −0.96, *p* < 0.05, for each CF concentration). A similarly high inverse correlation was observed between log CFU/mL and oxygen uptake rate, which in turn was closely related to specific growth rate. Apparently, mutants with higher metabolic activity when exposed to CF receive more DNA damage, which complicates their repair when resuming growth on agar plates and, ultimately, leads to a decrease in CFU.

The greatest resistance to ciprofloxacin was demonstrated by the *cysB* mutant, whose log CFU/mL was two to three orders of magnitude higher than that in the parental strain at antibiotic concentrations of 0.3–10 μg/mL. The *mstA* mutant, which was previously reported to have increased sensitivity to various antibiotics, including quinolones [16], did not differ from the parent. Since this mutant produced a similar amount of H_2_S as the wt strain, the cause of restoration of antibiotic tolerance could be a frequently occurring suppressor mutation in the *ycjW* gene, which restores the ability of Δ*mstA* to produce H_2_S [38]. It was previously reported that the *cysK* mutant of *S. typhimurium*, which produces large amounts of H_2_S, has increased resistance to the fluoroquinolone ofloxacin when grown in M9 medium with sulfate [53]. Under our conditions, the effect of ciprofloxacin on the survival of the *cysK* mutant of *E. coli* depended on the dose of the antibiotic: number of colonies was 2.5 times greater at 0.3 μg/mL CF and 2 times less at 3 μg/mL CF compared to the wt strain. The effect of the studied mutations on bacterial survival was best expressed in the rapid killing phase under the influence of 0.3 μg/mL CF. At this CF concentration, the killing rate of the *tnaA* mutant was 1.4 times higher, and that of the *tcyP*, *gshA*, and *cysB* mutants was 1.2, 1.9, and 2.2 times lower compared to the parent. There was no statistically significant relationship between killing rate, intracellular cysteine concentration, and H_2_S production. However, a high direct correlation was found between killing rate and concentrations of GSH_in_ and GSH_out_ (*p* = 0.8, *p* < 0.05). This indicated that excess cysteine, some of which was incorporated into glutathione, could contribute to the increased lethal activity of low doses of the antibiotic. The decrease or absence of H_2_S production in the studied mutants did not guarantee increased sensitivity to ciprofloxacin, although at similar μ values, the *gshA* mutant, which produces H_2_S, was more tolerant, and *tnaA*, which formed little H_2_S, was more sensitive to 0.3 μg/mL CF than the parental strain.

## 4. Materials and Methods

### 4.1. Bacterial Strains and Growth Conditions

A parental strain of *Escherichia coli* BW25113 (wt) and single-knockout mutants used in this study were from the Keio collection [54] (Table S1). AN2343 (*cydD*1) was a generous gift from Dr. R. Poole [55]. The parental and mutant strains carrying transcriptional gene fusions *katG*::*lacZ* [56], *sulA*::*lacZ* [57], and *iucC*::*lacZ* [31] were created by transformation and PI transduction methods.

Bacteria were cultured in Luria–Bertani medium (LB Miller, Tissue culture, VWR Life Science, Solon, OH, USA). About 1 mL of the overnight culture was transferred to 80 mL of fresh medium to an initial OD_600_ of 0.05 and grown in 250 mL flasks with shaking (150 rpm) at 37 °C. Antibiotics chloramphenicol (Cam) (25 μg/mL) or ciprofloxacin (CF) (0.03, 0.3, 3, and 10 μg/mL) were added when OD_600_ reached 0.4. The specific growth rate (µ) was calculated by equation µ = ΔlnOD_600_/Δt, where t is the time in hours.

### 4.2. Real-Time Monitoring of Dissolved Oxygen (dO_2_), pH, and Sulfide

Dissolved oxygen and pH in *E. coli* cultures were continuously measured directly in the flasks using a Clarke oxygen electrode InPro 6800 (Mettler Toledo, Greifensee, Switzerland) and a pH electrode ESC-10601/7 (“IT”Company, Moscow, Russia), respectively. The dO_2_/pH controller of a BioFlo 110 fermentor (New Brunswick Scientific Co., Edison, NJ, USA) was used for data recording.

Extracellular sulfide was continuously recorded directly in the flasks using the system of sulfide-specific ion-selective chalcogenide XC-S2-001 (operating pH range 6–12) (Sensor Systems Company, St. Petersburg, Russia) and reference electrodes and a computer pH/ion meter cpX-2 (IBI, Pushchino, Russia). The sulfide concentration in the medium was calculated using a standard curve prepared with known amounts of Na_2_S. However, it should be taken into account that during the experiments part of the hydrogen sulfide is lost to the gas phase. The synchronous processing of all primary data from the sensor system was carried out using the RS-232 and Modbus protocols and the Advantech OPC Server v3.0 software package.

### 4.3. Determination of H_2_S, Cysteine, and Glutathione

The formation of gaseous H_2_S was assessed using lead acetate [Pb(Ac)_2_], which reacts specifically with H_2_S to form a brown lead sulfide stain. Lead acetate-soaked paper strips were affixed in culture flasks above the surface of the liquid culture. For kinetic experiments, paper strips were sequentially replaced every 30 min. To determine the total H_2_S, the paper strip was left for 2 h. The spots were scanned and then quantified using ImageJ 1.54g. All experiments were performed 3–6 times on separate days.

For intracellular L-cysteine assays, 40 mL of culture (20 mL each from two identical flasks) were centrifuged (8000× *g* for 5 min), suspended in 4 mL of 0.1 M Tris-HCl pH 8.6, and lysed by sonification at 0 °C, using a 30 s pulse for six cycles. Perchloric acid (the final concentration 0.5 M) was added to the lysate to precipitate proteins. After 30 min, the suspension was centrifuged (8000× *g* for 5 min) and the supernatant was adjusted to pH 8.6 with KOH, frozen, centrifuged to eliminate the potassium perchlorate, and evaporated using a rotary evaporator RV10 (IKA, Staufen, Germany) at 65 °C to 0.57 mL and then treated with 0.25 mL of dithiothreitol (100 mM) for 10 min. Reduced samples (0.5 mL) were used to determine the amount of cysteine according to the Gaitonde method [58]. Standard curves were prepared with known amounts of cysteine, which were treated as samples of cell suspensions.

To determine intracellular total reduced and oxidized glutathione (GSH + GSSG), 5 mL cell culture samples were harvested by centrifugation (8000× *g* for 5 min) at 30 min intervals, resuspended in 5 mL of cold 20 mM EDTA and lysed by sonification at 0 °C, using a 30 s pulse for six cycles. Then, protein was precipitated as for the intracellular cysteine assay and glutathione was measured using the 5,5’-dithio-bis-(2-nitrobenzoic acid) (DTNB)-glutathione reductase recycling method, as described previously [59]. Standard curves were prepared with known amounts of GSH, which were treated as cell suspension samples.

Extracellular glutathione was determined as described previously [59] in samples that were removed at 30 min intervals by rapid filtration through 0.45 μm-pore-size membrane filters. Because LB medium contains glutathione, glutathione content in cell-free medium was subtracted from the obtained values to determine the GSH exported by the cells.

### 4.4. Study of Cellular Viability and Gene Expression

Minimum inhibitory concentrations (MICs) were determined by the microdilution method as outlined by the Clinical and Laboratory Standards Institute [60]. LB medium was used to grow cells. After 22 h of incubation at 37 °C, the cell density (OD_600_) in each well of the plates was measured using the 96-well microplate spectrophotometer xMark™ “Bio-Rad”. Experiments were conducted three times on separate days.

For colony-forming studies, culture samples were washed, diluted in 0.9% NaCl, mixed with molten soft LB-agar (0.8%) at 42 °C, and poured onto plates with solid LB-agar (1.5%). Colonies were counted over 24 h incubation at 37 °C. The rates of bacterial killing induced by ciprofloxacin were calculated using equation ψ = ln(N_t_/N_0_)/t, where N_t_ is the cell density (CFU/mL) at time t, N_0_ the initial cell density, and t the time in hours [61].

Changes in the expression of the tested genes were estimated by the determination of β-galactosidase activity [62] in *E. coli* strains carrying the appropriate gene fusions.

### 4.5. Statistical Analysis of the Data

Each result is indicated as the mean value of three to five independent experiments ± the standard error of the mean (SEM). Significant difference was analyzed by Student’s *t*-test. A *p*-value of 0.05 was used as the cut-off for statistical significance. Results were analyzed by means of program packet Statistica 8.0.360 (StatSoft Inc., Tulsa, OK, USA, accessed on 27 August 2007).

## 5. Conclusions

The production of H_2_S by *E. coli* cells during growth in LB medium is a consequence of triggering cysteine homeostasis mechanisms to prevent the accumulation of excessive cysteine in the cytoplasm during the active import of cystine from the medium. Mutations in the cystine (*tcyP*) and cysteine (*eamA*) transport systems, cysteine degradation enzymes (*mstA*, *cysK*, *tnaA*, *cyuA*), GSH synthesis (*gshA*), and cysteine regulon regulator (*cysB*) affected H_2_S production, growth and respiration rates and caused significant changes in the content of cysteine and glutathione and the degree of induction of Fur and SOS regulons. Exposure to chloramphenicol and high doses of ciprofloxacin increased intracellular cysteine concentrations and stimulated the incorporation of excess cysteine into glutathione, indicating a disturbance in cysteine homeostasis. Increased tolerance to ciprofloxacin in *tcyP*, *gshA*, and *cysB* mutants could be due to a limited rate of cystine transport into these cells. Sensitivity to ciprofloxacin could apparently be influenced by the entire set of physiological changes caused by mutations, including changes in the redox situation and the induction of protective systems. Over a wide range of ciprofloxacin concentrations, a high inverse relationship was revealed between log CFU/mL of the studied mutants and the specific growth rate of bacteria in LB medium before the addition of the antibiotic. The study points to the important role of maintaining cysteine homeostasis for bacterial fitness during normal growth and response to antibiotics. Our further research is aimed at studying the role of cysteine homeostasis in antibiotic therapy of infections caused by pathogenic strains of *E. coli*, including multidrug-resistant avian pathogens (APEC).

## Data Availability

Data used to support the findings of this study are available from the corresponding author upon reasonable request.

## Supplementary Materials

The following supporting information can be downloaded at: https://www.mdpi.com/article/10.3390/ijms25084424/s1.
