# Supplementary material for: Regulation of Cysteine Homeostasis and Its Effect on Escherichia coli Sensitivity to Ciprofloxacin in LB Medium
Source: Int J Mol Sci. 2024 Apr 17;25(8):4424. doi: 10.3390/ijms25084424 (PMC11050555; doi:10.3390/ijms25084424)
Supplement: Supplementary file 1 [file ijms-25-04424-s001.zip › Figure S2.pdf]

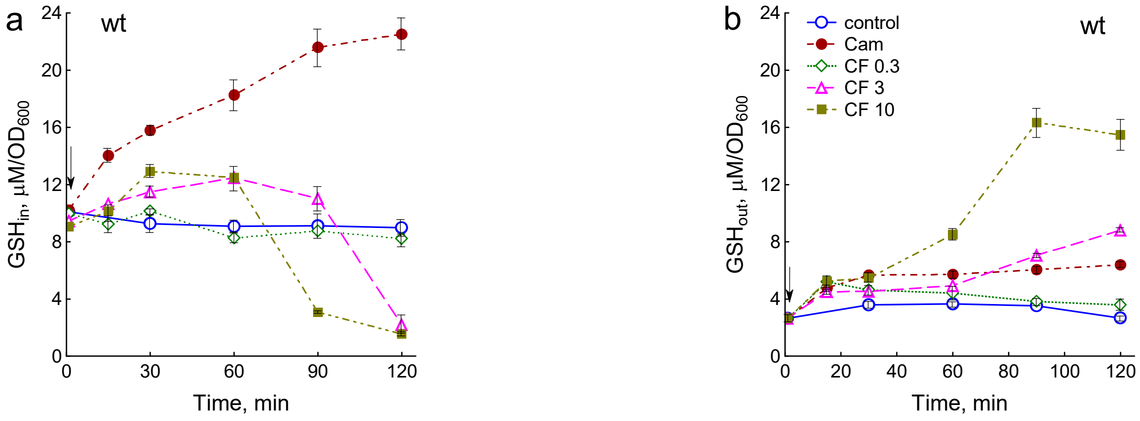

Figure S2. Changes in intracellular (GSH_{in}) (**a**) and extracellular (GSH_{out}) (**b**) glutathione when *E. coli* BW25113 (wt) was treated with chloramphenicol (Cam 25 µg/ml) or ciprofloxacin (CF 0.3-10 µg/ml).
