# Supplementary material for: Regulation of Cysteine Homeostasis and Its Effect on Escherichia coli Sensitivity to Ciprofloxacin in LB Medium
Source: Int J Mol Sci. 2024 Apr 17;25(8):4424. doi: 10.3390/ijms25084424 (PMC11050555; doi:10.3390/ijms25084424)
Supplement: Supplementary file 1 [file ijms-25-04424-s001.zip › Figure S3.pdf]

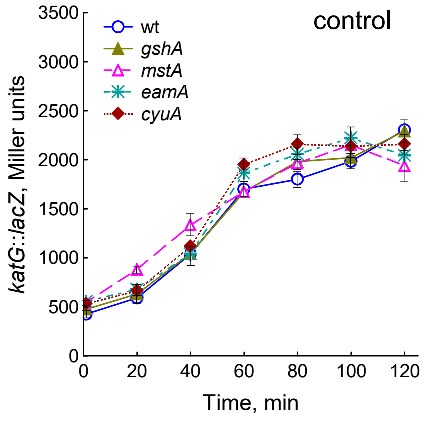

Figure S3. The expression of *katG*::*lacZ* in the *gshA*, *mstA*, *eamA*, and *cyuA* mutants does not differ significantly from the parental strain.
