# Supplementary material for: Regulation of Cysteine Homeostasis and Its Effect on Escherichia coli Sensitivity to Ciprofloxacin in LB Medium
Source: Int J Mol Sci. 2024 Apr 17;25(8):4424. doi: 10.3390/ijms25084424 (PMC11050555; doi:10.3390/ijms25084424)
Supplement: Supplementary file 1 [file ijms-25-04424-s001.zip › Figure S6.pdf]

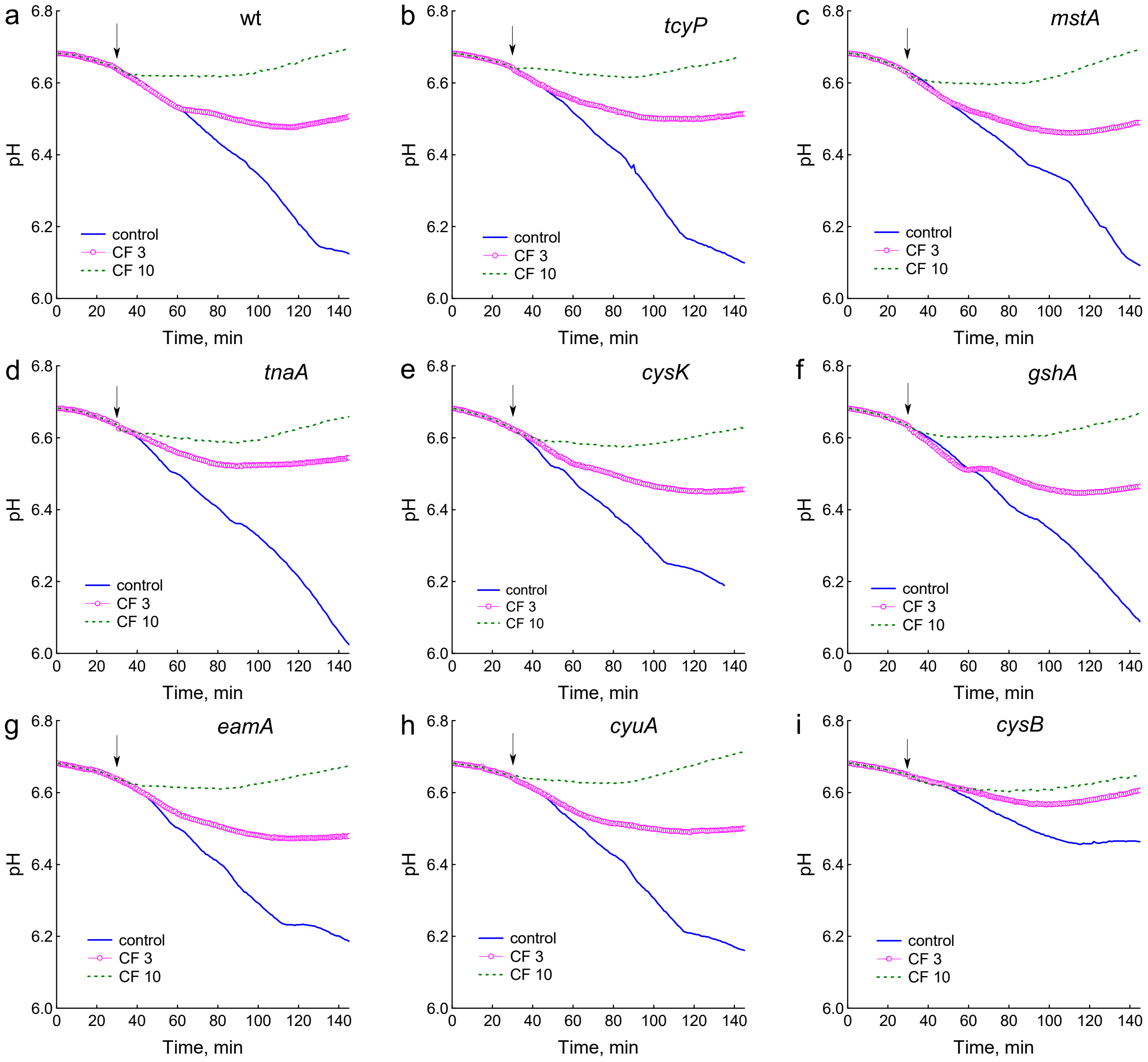

Figure S6. Effect of ciprofloxacin (3 and 10 µg/ml) on pH changes in the culture medium of the studied *E. coli* strains. The time for adding ciprofloxacin is indicated by the arrow.
