# Supplementary material for: Regulation of Cysteine Homeostasis and Its Effect on Escherichia coli Sensitivity to Ciprofloxacin in LB Medium
Source: Int J Mol Sci. 2024 Apr 17;25(8):4424. doi: 10.3390/ijms25084424 (PMC11050555; doi:10.3390/ijms25084424)
Supplement: Supplementary file 1 [file ijms-25-04424-s001.zip › Figure S7.pdf]

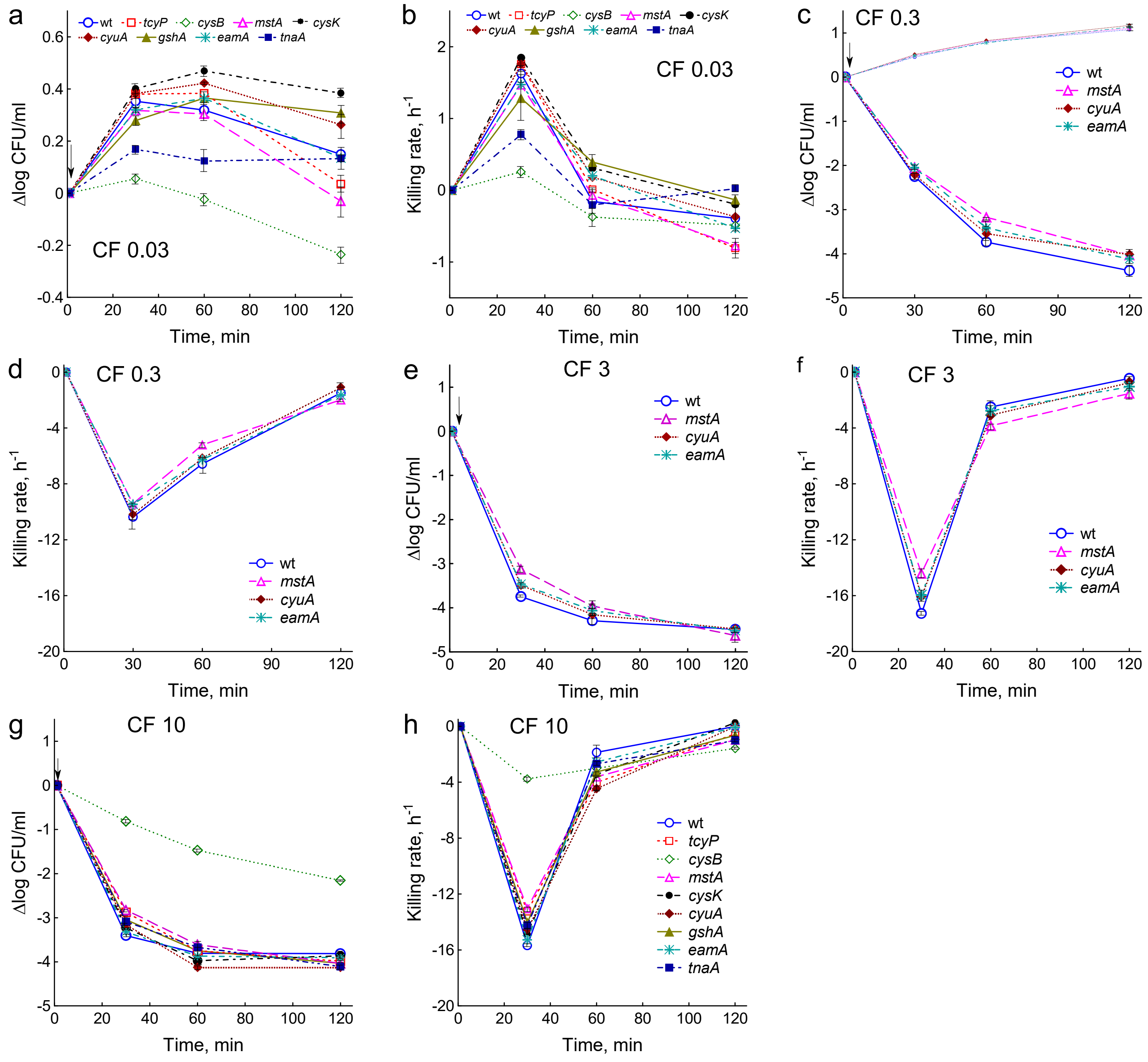

Figure S7. Killing curves (**a**,**c**,**e**,**g**) and killing rate (**b**,**d**,**f**,**h**) upon exposure of studied *E. coli* mutants to 0.03 (**a**,**b**), 0.3 (**c**,**d**), 3 (**e**,**f**) and 10 µg/ml (**g**,**h**) ciprofloxacin. The time for adding ciprofloxacin is indicated by the arrow. Values are the means and standard error (vertical bars) from at least three independent experiments.
