# Supplementary material for: Regulation of Cysteine Homeostasis and Its Effect on Escherichia coli Sensitivity to Ciprofloxacin in LB Medium
Source: Int J Mol Sci. 2024 Apr 17;25(8):4424. doi: 10.3390/ijms25084424 (PMC11050555; doi:10.3390/ijms25084424)
Supplement: Supplementary file 1 [file ijms-25-04424-s001.zip › Table S1.pdf]

Table S1. *Escherichia coli* strains and plasmids used in this study.

Strain	Genotype	Source
BW25113	*Δ(araD-araB)567*, *ΔlacZ4787*(::rrnB-3), λ⁻, *rph-1*, *Δ(rhaD-rhaB)568*, *hsdR514*	Baba et al., 2006 [1]
JW2663	as BW25113, but Δ*gshA*	Baba et al., 2006 [1]
JW5250	as BW25113, but Δ*eamA*	Baba et al., 2006 [1]
JW2562	as BW25113, but Δ*eamB*	Baba et al., 2006 [1]
JW5363	as BW25113, but Δ*bcr*	Baba et al., 2006 [1]
JW2407	as BW25113, but Δ*cysK*	Baba et al., 2006 [1]
JW2414	as BW25113, but Δ*cysM*	Baba et al., 2006 [1]
JW2505	as BW25113, but Δ*mstA* (*sseA*)	Baba et al., 2006 [1]
JW1614	as BW25113, but Δ*malY*	Baba et al., 2006 [1]
JW2975	as BW25113, but Δ*metC*	Baba et al., 2006 [1]
JW5518	as BW25113, but Δ*cyuA* (*yhaM*)	Baba et al., 2006 [1]
JW2514	as BW25113, but Δ*iscS*	Baba et al., 2006 [1]
JW3686	as BW25113, but Δ*tnaA*	Baba et al., 2006 [1]
JW1267	as BW25113, but Δ*cysB*	Baba et al., 2006 [1]
JW0437	as BW25113, but Δ*cyuR* (*ybaO*)	Baba et al., 2006 [1]
JW1718	as BW25113, but Δ*tcyP* (*ydjN*)	Baba et al., 2006 [1]
AN2342	K-12 derivative, F-	Cruz-Ramos et al., 2004 [2]
AN2343	as AN2342 *cydD1*, G319D, G429E	Cruz-Ramos et al., 2004 [2]
DM4000	*hisG4 argE3 thr-1- ara-14 xyl-5 mtl-1 rpsL31 tsx-33 ilv TS sulA::Mud1(bla lac) cam*	Volkert et al. 1989 [3]
	Plasmids	
pKT1033	*katG*::*lacZ*	Tao et al. 1989 [4]
pColV-K30	*iucC::lacZ*	Maringanti and Imlay, 1999 [5]

References

1. Baba, T.; Ara, T.; Hasegawa, M.; Takai, Y.; Okumura, Y.; Baba, M.; Datsenko, K.A.; Tomita, M.; Wanner, B.L.; Mori, H. Construction of *Escherichia coli* K-12 in-frame, single-gene knockout mutants: The Keio collection. *Mol. Syst. Biol.* **2006**, *2*, 2006.0008. https://doi.org/10.1038/msb4100050.
2. Cruz-Ramos, H.; Cook, G.M.; Wu, G.; Cleeter, M.W.; Poole, R.K. Membrane topology and mutational analysis of *Escherichia coli* CydDC, an ABC-type cysteine exporter required for cytochrome assembly. *Microbiology* **2004**, *150*, 3415–3427. https://doi.org/10.1099/mic.0.27191-0.
3. Volkert, M.R.; Gately, F.H.; Hajec, L.I. Expression of DNA damage-inducible genes of *Escherichia coli* upon treatment with methylating, ethylating and propylating agents. *Mutat. Res.* **1989**, *217*, 109–115. https://doi.org/10.1016/0921-8777(89)90062-1.
4. Tao, K.; Makino, K.; Yonei, S.; Nacata, A.; Shinagawa, H. Molecular cloning and nucleotide sequencing of *oxyR*, the positive regulatory gene of a regulon for an adaptive response to oxidative stress in *Escherichia coli*: Homologies between OxyR protein and a family of bacterial activator proteins. *Mol. Gen. Genet*. **1989**, *218*, 371–376. https://doi.org/10.1007/bf00332397.
5. Maringanti, S.; Imlay, J.A. An intracellular iron chelator pleiotropically suppresses enzymatic and growth defects of superoxide dismutase-deficient *Escherichia coli*. *J. Bacteriol.* **1999**, *181*, 3792–3802. https://doi.org/10.1128/JB.181.12.3792-3802.1999.
