# Supplementary material for: Regulation of Cysteine Homeostasis and Its Effect on Escherichia coli Sensitivity to Ciprofloxacin in LB Medium
Source: Int J Mol Sci. 2024 Apr 17;25(8):4424. doi: 10.3390/ijms25084424 (PMC11050555; doi:10.3390/ijms25084424)
Supplement: Supplementary file 1 [file ijms-25-04424-s001.zip › Figure S1.pdf]

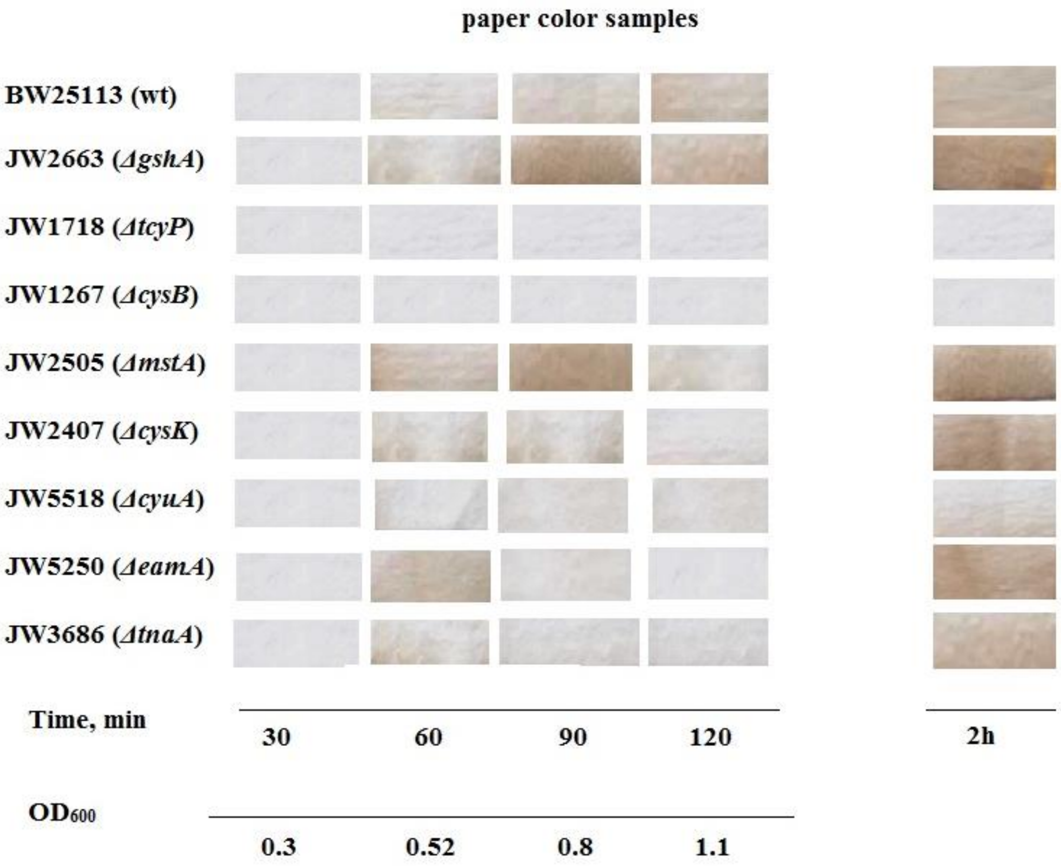

Figure S1. H2S accumulation in the gas phase of E. coli wt and mutant strains. Shown are samples of lead acetate-soaked paper strips after successive 30-minute exposures (left) or 2-hour exposure (right) over the surface of cultures growing in LB medium. For each time point, the corresponding value of the optical density of the culture (OD600) is indicated.
