# Supplementary figures and images for: Regulation of Cysteine Homeostasis and Its Effect on Escherichia coli Sensitivity to Ciprofloxacin in LB Medium
Source: Int J Mol Sci. 2024 Apr 17;25(8):4424. doi: 10.3390/ijms25084424 (PMC11050555; doi:10.3390/ijms25084424)

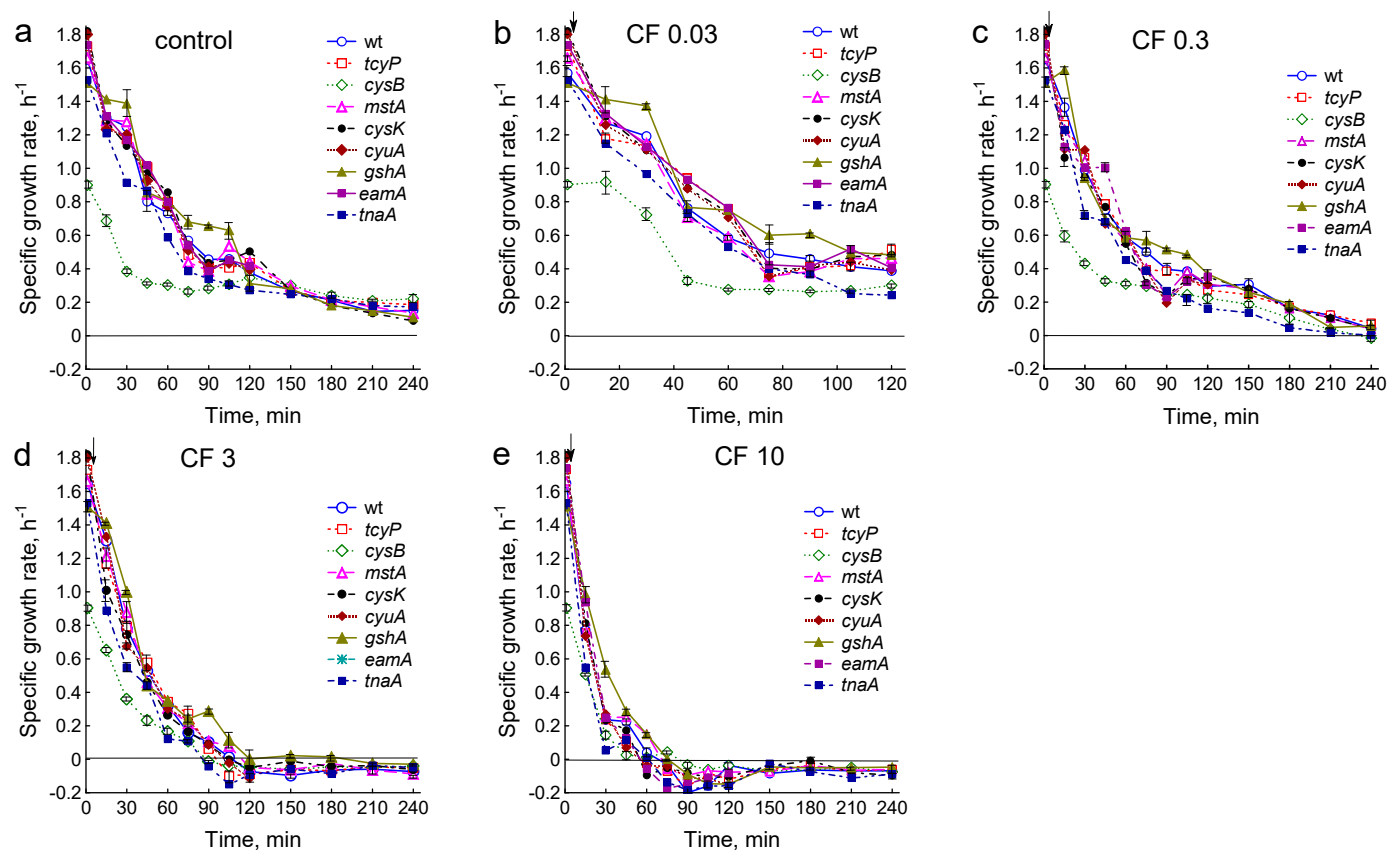

**Figure S4.** Effect of ciprofloxacin on the specific growth rate of the studied mutants.

Supplement: Supplementary file 1 [file ijms-25-04424-s001.zip › Figure S4.pdf]
